# Treatment benefit in patients aged 80 years or older with biopsy-proven and non-resected glioblastoma is dependent on *MGMT* promoter methylation status

**DOI:** 10.1007/s11060-023-04362-y

**Published:** 2023-06-08

**Authors:** Jonathan Weller, Sophie Katzendobler, Sebastian Niedermeyer, Patrick N. Harter, Jochen Herms, Christoph Trumm, Maximilian Niyazi, Niklas Thon, Joerg-Christian Tonn, Veit M. Stoecklein

**Affiliations:** 1 Department of Neurosurgery, University Hospital, LMU Munich, Marchioninistrasse 15, Munich, 81377 Germany; 2 Center for Neuropathology and Prion Research, LMU Munich, Munich, Germany; 3 Department of Neuroradiology, LMU Munich, Munich, Germany; 4 Department of Radiotherapy and Radiation Oncology, University Hospital, LMU Munich, Munich, Germany; 5 German Cancer Consortium (DKTK), Partner site Munich and German Cancer Research Center (DKFZ), Heidelberg, Germany

**Keywords:** Glioblastoma, Elderly, Frailty, Temozolomide, Radiotherapy, MGMT

## Abstract

**Purpose:**

Glioblastoma is associated with especially poor outcome in the elderly. It is unclear if patients aged **≥**80 years benefit from tumor-specific therapy as opposed to receiving best supportive care (BSC) only.

**Methods:**

Patients with IDH-wildtype glioblastoma (WHO 2021), aged **≥**80 years, and diagnosed by biopsy between 2010 and 2022 were included. Patient characteristics and clinical parameters were assessed. Uni- and multivariate analyses were performed.

**Results:**

76 patients with a median age of 82 (range 80–89) and a median initial KPS of 80 (range 50–90) were included. Tumor-specific therapy was initiated in 52 patients (68%). 22 patients (29%) received temozolomide monotherapy, 23 patients (30%) were treated with radiotherapy (RT) alone and 7 patients (9%) received combination therapies. In 24 patients (32%), tumor-specific therapy was omitted in lieu of BSC. Overall survival (OS) was longer in patients receiving tumor-specific therapy (5.4 vs. 3.3 months, p < 0.001). Molecular stratification showed that the survival benefit was owed to patients with MGMT promoter methylation (*MGMT*pos) who received tumor-specific therapy as opposed to BSC (6.2 vs. 2.6 months, p < 0.001), especially to those with better clinical status and no initial polypharmacy. Patients with unmethylated *MGMT* promoter (*MGMT*neg) did not benefit from tumor-specific therapy (3.6 vs. 3.7 months, p = 0.18). In multivariate analyses, better clinical status and *MGMT* promoter methylation were associated with prolonged survival (p < 0.01 and p = 0.01).

**Conclusion:**

Benefit from tumor-specific treatment in patients with newly diagnosed glioblastoma aged **≥**80 years might be restricted to *MGMT*pos patients, especially to those with good clinical status and no polypharmacy.

**Supplementary Information:**

The online version contains supplementary material available at 10.1007/s11060-023-04362-y.

## Introduction

Glioblastomas are highly aggressive primary tumors of the central nervous system. The combination of an isocitrate dehydrogenase (IDH) wildtype status with at least one of the following molecular alterations defines a glioblastoma: EGFR amplification, combined chromosome 7 gain and chromosome 10 loss or TERT promoter mutation [1–3]. The median patient age at diagnosis is 64 years and age-standardized incidence rates of glioblastoma are rising in western societies across all age groups [4–8]. Life expectancy in patients with glioblastoma aged older than 60 years is less than 12 months as higher patient age is a major factor compromising survival [9–13]. Available treatment options for glioblastoma are potentially toxic and exhausting. Patients in the highest age bracket, aged 80 years and older, are especially vulnerable to these treatment side effects because of a higher incidence of pre-existing morbidity and increasing frailty. This gives rise to the question, whether these oldest-old patients benefit from tumor-specific treatment at all or whether the toxic effects of treatment outweigh potential survival benefits and best supportive care should be preferred.

This question appears even more pressing as the proportion of elderly people is rising on a population-based level. According to the World Health Organization, the overall number of people over the age of 80 is expected to triple by the year 2050 [14]. Many who reach this age maintain a high quality of life and high levels of activity and independence [15]. This means that health care providers are faced with a rising number of oldest-old patients with glioblastoma and good functional status. Yet, evidence-based treatment guidelines in this scenario are scarce. This is because the elderly are vastly underrepresented in clinical trials due to various reasons, e.g., inclusion criteria that include age limits, comorbidities, decisions against therapy made both by patients and families as well as the fact that caregivers are hesitant to enroll elderly patients in clinical trials [16–19].

It was our aim to investigate benefit of tumor-specific therapy versus best supportive care (BSC) in the oldest-old and determine subgroups eligible for tumor-specific treatment while accounting for clinical status, molecular parameters, initial tumor volumes and localizations. We furthermore included the temporal muscle thickness (TMT) in our analyses, a surrogate for sarcopenia that has been described as a prognostic marker in glioblastoma [20, 21]. We sought to investigate the role of the O6-methylguanine-DNA methyltransferase (*MGMT)* promoter methylation status, an important prognostic and predictive marker for response to the alkylating chemotherapy agent temozolomide, as a potential stratifier for therapy in the elderly as proposed by prospective, clinical trials in the past [10, 12, 22, 23].

## Patients and methods

### Patient evaluation

The institutional database was screened retrospectively for patients with newly diagnosed glioblastoma, IDH-wildtype CNS WHO grade 4, between 2010 and 2022 after approval by the local ethics committee (project number 23–0052). Further patient inclusion criteria were as follows: (I) patient age of 80 years or older at first diagnosis, (II) diagnosis through stereotactic biopsy and (III) an integrated histopathological and molecular diagnosis according to the WHO 2021 Classification of Tumors of the Central Nervous System. Patient-related and clinical parameters such as patient age, sex, clinical status according to the Karnofsky Performance Status (KPS), date of biopsy and medication were assessed. The number of prescribed medications taken by individual patients was assessed as polypharmacy is associated with frailty [24]. The medication had to be prescribed by a physician and administered orally or intravenously or intramuscularly on a regular schedule to be included. Progression-free (PFS) and overall survival (OS) were investigated as outcome measures. PFS was defined according to RANO (Response Assessment in Neuro-Oncology) criteria [25]. OS was defined as the time interval from first diagnosis to the date of glioblastoma-related death. When the exact date and cause of death were missing in the local databases, e.g., due to initiation of palliative care in an hospice, respective general practitioners were contacted. Exact dates and thus OS could be obtained in all patients who died. 2 patients were alive at data base closure.

### Histopathology and molecular analyses

Histological sampling was achieved in all patients through a stereotactic, frame-based biopsy technique [26–28]. Histopathological and molecular analyses were conducted at the institutional neuropathology. An integrated diagnosis according to WHO 2021 was provided in all patients. By determining methylation status of 25 CpG sites in the *MGMT* promoter region through sequencing of sodium-bisulfite-modified DNA, *MGMT* promoter methylation was assessed [29]. For exclusion of isocitrate dehydrogenase gene 1 or 2 mutations, pyrosequencing for detection of hotspot mutations was performed.

### Tumor volumes and temporal muscle thickness

Initial tumor volume on T2 weighted and T1 weighted, contrast-enhanced magnetic resonance imaging (MRI) scans were manually segmented. To this end, Brainlab Elements Smartbrush software by Brainlab (Brainlab AG, Munich, GER) was utilized. The volume of pathological contrast-enhancement on initial MRI is hereafter referred to as CE volume. TMT was evaluated on T1-weighted scans and according to previously published literature [20, 21, 30, 31].

### Therapy

Treatment choices were evaluated in interdisciplinary tumor boards for all patients. Recommendations were discussed with the patients and treatment was initiated based on patients’ and caregivers’ preferences. Temozolomide was given at a daily dose of 75 mg/m^2^ concomitantly to radiotherapy if applicable and in case of temozolomide alone at a dose of 150-200 mg/m^2^ in a 5/28 schedule. Total dose of involved-field radiotherapy was planned with either 40.05 Gy (hypofractionated) or 60 Gy (standard radiotherapy). Treatment was discontinued in case of treatment-related severe adverse events, if patients wished to stop tumor-specific therapy or in case of clinical deterioration. Complications from therapy were assessed in accordance with the Common Terminology Criteria for Adverse Events (CTCAE) version 5 [32]. BSC was defined as palliative care without any tumor-specific therapy and comprised physical, social and psychological support as well as prescription of anticonvulsive medication, analgesics and corticosteroids if deemed necessary.

### Statistical analysis and matched-pair analysis

Descriptive and comparative statistics were done with GraphPad PRISM 9.4.1 software. Normal distribution and variance were calculated by D’Agostino-Pearson test. Student’s t-test was conducted to assess differences between two groups and ANOVA for multiple groups in case of parametric data. For nonparametric data, Mann-Whitney U-test and Kruskal-Wallis tests were conducted. Comparative testing of categorial variables was done by chi-squared test. Univariate analyses of categorial variables were performed through Kaplan-Meier estimate and logrank tests. Additionally, Cox proportional hazards models were used for continuous and categorical variables. For multivariate analysis, Cox proportional hazards models were used. The validity of the proportional hazards assumption was tested by determining scaled Schoenfeld residuals versus time. Hazard ratios (HR) and 95% confidence interval (95% CI) of HRs were calculated. Statistical significance was assumed for p ≤ 0.05. A matched-pair analysis accounting for KPS and CE volume was conducted after identifying clinical status and initial CE volumes to be prognostic in univariate analysis (Table 1). Pairing of two CE values was deemed acceptable only if the tumor volume of one tumor did not exceed the other volume by more than 10% as described by other studies [27, 33].

**Table 1:** Univariate analyses for progression-free and overall survival

Factor		PFS			OS		
		HR	95% CI	*p*-value	HR	95% CI	*p*-value
**Age** ^**x**^		1.03	0.93–1.13	0.54	1.03	0.93–1.13	0.59
**KPS** ^**x**^		0.96	0.94–0.99	< 0.01*	0.96	0.95–0.98	< 0.01*
**Polypharmacy**		1.1	0.98–1.23	0.11	1.1	0.97–1.23	0.13
**T2 tumor volume** ^**x**^		1.01	1.0-1.02	0.04*	1.01	1.0-1.02	0.03*
**CE volume** ^**x**^		1.01	1.0-1.02	0.04*	1.01	1.0-1.02	0.03*
**Multifocal manifestation**		0.93	0.53–1.56	0.79	1.01	0.57–1.69	0.98
**Frontal versus non-frontal localization**		2.05	1.11–4.16	0.03*	1.71	0.94–3.37	0.1
**TMT** ^**x**^		1.04	0.91–1.19	0.55	1.05	0.93–1.19	0.42
***MGMT*** **promoter methylation status**		0.65	0.4–1.04	0.07	0.52	0.31–0.85	< 0.01*

KPS, Karnofsky Performance Status; CE volume, contrast-enhancing tumor volume; TMT, temporal muscle thickness; MGMT, O6-methylguanine-DNA methyltransferase; PFS, progression-free survival; OS, overall survival; ^x^ Continuously scaled. Significant p-values are highlighted with asterisks*

## Results

### Study population, clinical and imaging parameters and MGMT methylation status

Overall, 76 patients with a median age of 82 (range 80–89) and a median initial KPS of 80 (range 50–90) were included in the study (Table 2). The ratio of female to male patients was 0.7:1 (31:45). At diagnosis, multiple lobes were visibly affected on MRI in 28 patients (37%). Initial mean T2 tumor volume was 36.7 cm^3^ (standard deviation 29.4). Median TMT was 7.5 cm (range 3-12.2, see Table 2). No severe adverse event related to the stereotactic biopsy was seen.

**Table 2:** Patient characteristics

Parameter	All patients (n = 76)	Best supportive care (n = 24)	Treatment (n = 52)	*p*-value
Age (years)				
Median	82	82	82	*0.86*
Range	80–89	80–87	80–89	
**Sex, n (%)**				
Female	31 (41)	10 (42)	21 (40)	*0.92*
Male	45 (59)	14 (58)	31 (60)	
**KPS at first admission, n (%)**				
100	0 (0)	0 (0)	0 (0)	*0.1*
90	17 (22)	5 (21)	12 (23)	
80	35 (46)	7 (29)	28 (54)	
< 80	24 (32)	12 (50)	12 (23)	
***MGMT*** **promoter methylation status**				
methylated	38 (50)	8 (33)	30 (58)	0.05*
unmethylated	38 (50)	16 (67)	22 (42)	
**Localization, n (%)**				
Multilobular	28 (37)	12 (50)	16 (31)	*0.25*
Frontal	13 (17)	2 (8)	11 (21)	
Temporal	18 (24)	7 (29)	11 (21)	
Parietal	9 (12)	1 (4)	8 (15)	
Occipital	2 (3)	1 (4)	1 (2)	
Corpus callosum	4 (5)	0 (0)	4 (8)	
Midline	2 (3)	1 (4)	1 (2)	
**Laterality, n (%)**				
Left	40 (53)	9 (38)	31 (60)	*0.14*
Right	29 (38)	13 (54)	16 (31)	
Bilateral	7 (9)	2 (8)	5 (10)	
**T2 tumor volume (cm** ^**3**^ **)**				
Mean	36.7	48.5	31.2	0.03*
Standard deviation	29.4	34.4	25.3	
**CE volume (cm** ^**3**^ **)**				
Median	29.7	38.1	25.7	0.05*
Standard deviation	24.5	24.7	23.6	
**Temporal muscle thickness (mm)**				
Median	7.5	7.5	7.2	*0.73*
Range	3.3–12.2	4.9–12.2	3.3–11.3	

KPS, Karnofsky Performance Status; MGMT, O6-methylguanine-DNA methyltransferase; CE volume, contrast-enhancing tumor volume. Significant p-values are highlighted with asterisks*

### Treatment and adverse events

A total of 52 patients (68%) received tumor-specific therapy consisting of radiochemotherapy (n = 7; 9%), radiotherapy alone (n = 23; 30%) or temozolomide alone (n = 22; 29%). BSC without tumor-specific therapy was initiated in 24 patients (32%). In the radiotherapy cohort, 20/23 patients (87%) were treated with a hypofractionated irradiation regimen. In case of chemotherapy alone, median number of completed TMZ cycles was 3 (range 1–14). Therapy discontinuation rates were 43% (3/7) in the radiochemotherapy cohort, 64% (14/22) in the temozolomide cohort and 26% (6/23) in the radiotherapy cohort (Suppl. Table 1). Severe adverse events, i.e., CTCAE grade 3 or higher, occurred in 8 patients (15%) (Suppl. Table 1). There was no treatment-related fatal event.

In the treatment cohort, 30 patients (58%) showed a methylated *MGMT promoter (MGMT*pos) and 22 patients (42%) were *MGMT*neg. In the BSC cohort, 8 patients (33%) were *MGMTpos* and 16 patients (67%) *MGMT* neg. These differences were statistically significant (p = 0.05, see Table 2). A significant difference between the BSC and the therapy cohort was also seen in the size of initial T2 tumor volume and CE volume as those receiving therapy had a smaller mean tumor volume (therapy versus BSC; in cm^3^; median T2 volume: 31.2 versus 48.5, p = 0.03; mean CE volume: 25.7 versus 38.1, p = 0.05). Other potential prognostic factors such as clinical status, sex, age at diagnosis, TMT, site and multifocality of the tumor did not differ significantly between the two cohorts (Table 2).

### Progression-free and overall survival

**Fig. 1:**
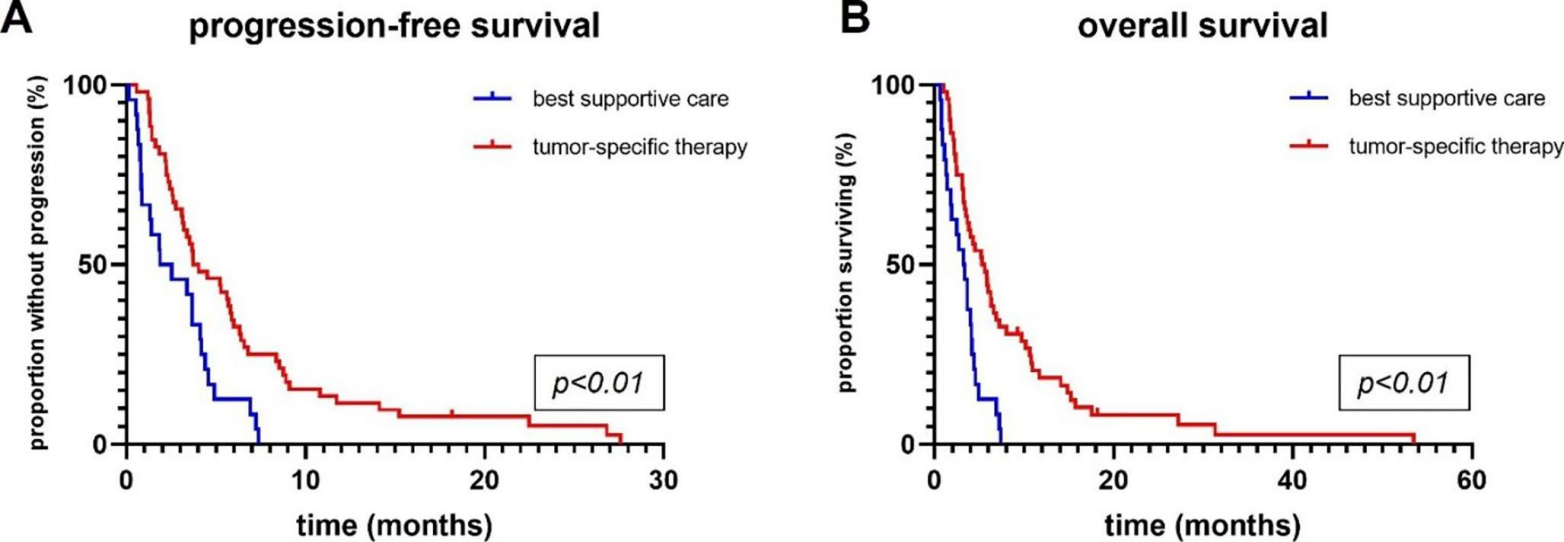
Kaplan-Meier estimates of progression-free survival (**A**) and overall survival (**B**) according to post-diagnostic strategy. Tumor-specific treatment was associated with longer progression-free and overall survival than best supportive care (p < 0.01). Treatments comprised temozolomide alone, radiotherapy alone and radiochemotherapy

**Fig. 2:**
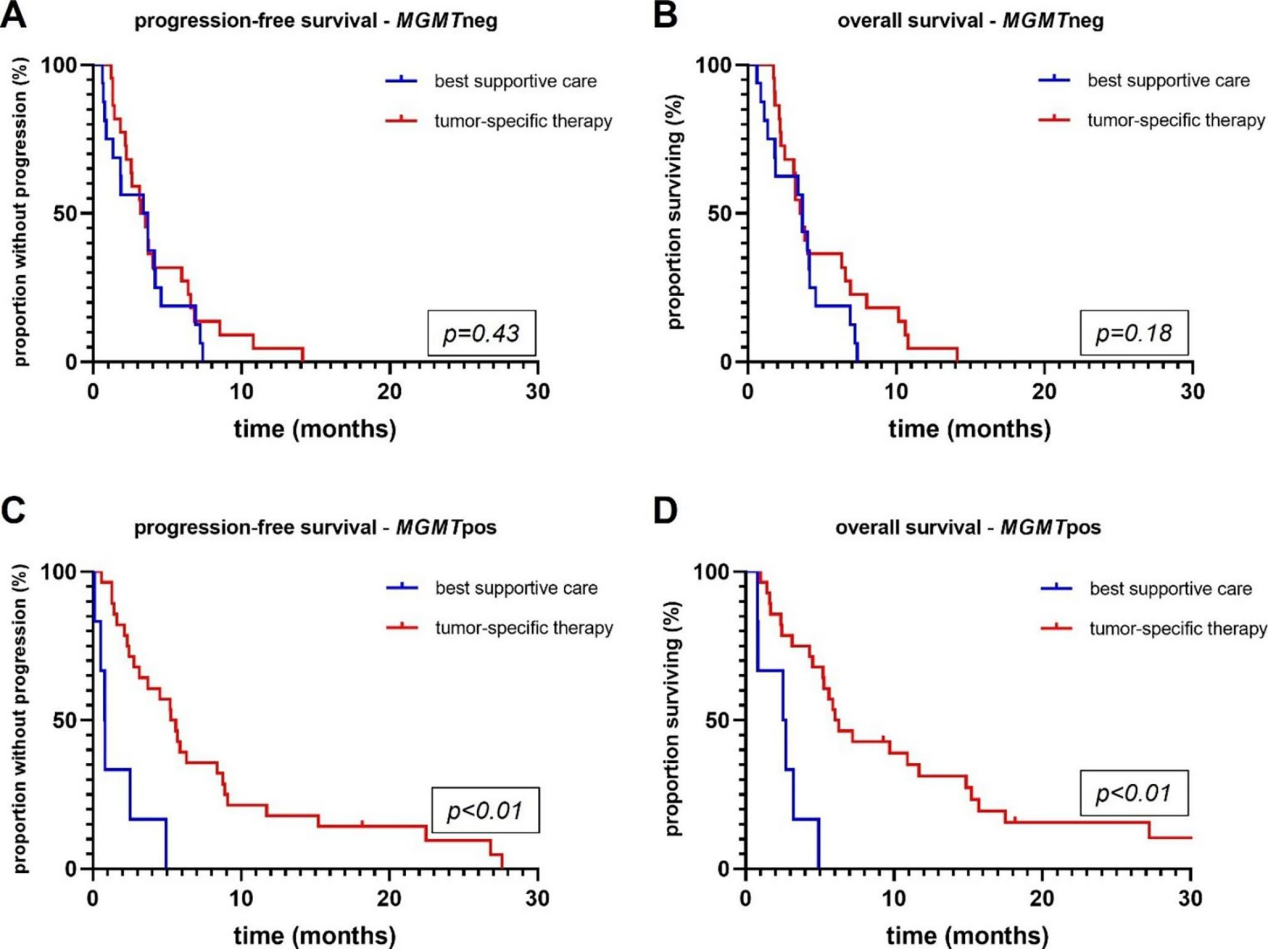
Kaplan-Meier estimates of progression-free (**A**, **C**) and overall survival (**B**, **D**) according to post-diagnostic strategy and stratified according to the *MGMT* (O6-methylguanine-DNA methyltransferase) promoter methylation status. Progression-free and overall survival was significantly longer in patients with *MGMT*pos tumors and receiving tumor-specific therapy when compared to best supportive care (p < 0.01) (**C**, **D**), but did not differ between the two cohorts in *MGMT*neg tumors (p = 0.43 and p = 0.18). Treatments comprised temozolomide alone, radiotherapy alone and radiochemotherapy. *MGMTpos*, methylated *MGMT* promoter; MGMTneg, unmethylated MGMT promoter

In the entire cohort, median PFS was 3.7 months and OS was 4.1 months. Patients with BSC showed a median OS of 3.3 as opposed to 5.4 months in patients who received treatment (logrank: tumor-specific therapy versus BSC; HR 2.34; 95% CI 1.28–4.28; p < 0.01, see Fig. 1). No patient primarily receiving BSC lived longer than 7.4 months. Stratifying patients according to their *MGMT* promoter methylation status showed that in *MGMT*neg tumors, there was no benefit of tumor-specific therapy over BSC regarding PFS (logrank: in months, 3.4 versus 3.5; HR 0.76; 95% CI 0.38–1.5; p = 0.43) or OS (logrank: in months, 3.6 versus 3.7; HR 0.61; 95% CI 0.3–1.26; p = 0.18) (Fig. 2). In *MGMT*pos glioblastomas, there was a strong association between prolonged PFS and OS in patients receiving tumor-specific therapy as opposed to BSC (logrank: tumor-specific therapy versus BSC; PFS: in months, 5.4 versus 0.8; HR 22.95; 95% CI 4.41–119.5; p < 0.01; OS: in months, 6.2 versus 2.6, HR 17.0, 95% CI 3.44–83.94; p < 0.01) (Fig. 2).

In the cohort of patients with *MGMT*pos tumors receiving therapy, 5 patients (5/30; 17%) lived past 18 months and 3 patients (3/30; 10%) were alive more than 2 years after diagnosis. A comparison between these cases and patients who died before 6 months despite receiving therapy (n = 15/30; 50%) demonstrated significantly better clinical status with a mean KPS of 86 versus 78 (p = 0.03) and less medication (mean number of medications, p = 0.01) at initial diagnosis in patients with more than 18 months survival. There was a trend towards smaller CE volumes in the cohort with longer survival with a mean CE volume of 9 cm^3^ versus 23 cm^3^ (p = 0.06). Age (p = 0.52), T2 tumor volume (p = 0.13), TMT (p = 0.98) and sex (p = 0.29) did not differ signicantly between the groups.

**Fig. 3:**
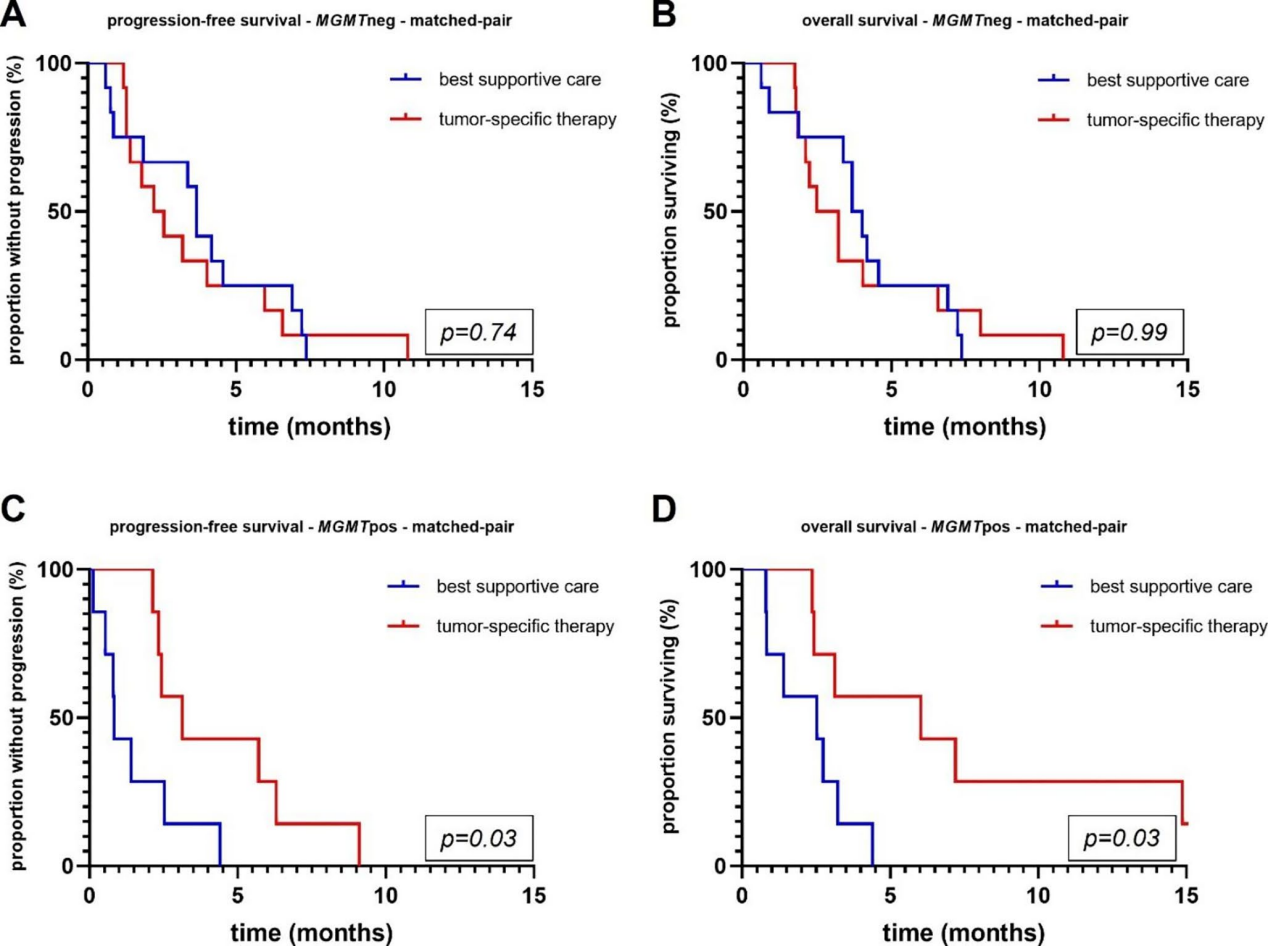
Kaplan-Meier estimates of progression-free (**A**, **C**) and overall survival (**B**, **D**) in a matched-pair analysis accounting for clinical status and initial CE volume and according to post-diagnostic strategy. The cohorts were stratified according to the *MGMT* (O6-methylguanine-DNA methyltransferase) promoter methylation status. Progression-free and overall survival was significantly longer in patients with *MGMT*pos tumors and receiving tumor-specific therapy when compared to best supportive care (p < 0.03) (C, D), but did not differ between the two cohorts in *MGMT*neg tumors (p = 0.74 and p = 0.99). Treatments comprised temozolomide alone, radiotherapy alone and radiochemotherapy. *MGMTpos*, methylated *MGMT* promoter; MGMTneg, unmethylated *MGMT* promoter; CE volume, contrast-enhancing tumor volume

In a matched-pair analysis that compared BSC with tumor-specific therapy and accounted for clinical status and CE volume, similar results were seen: PFS and OS did not differ significantly between the 12 *MGMT*neg pairs that were identified (PFS: in months, 3.7 versus 2.4, p = 0.74; OS: in months, 3.8 versus 2.8, p = 0.99). In the *MGMT*pos cohort, 7 pairs were identified. PFS and OS were significantly shorter in patients receiving BSC (PFS: in months, 0.8 versus 3.1, p = 0.03; OS in months, 2.5 versus 6.0, p = 0.03, see Fig. 3). Mean CE tumor volume of patients treated with BSC was 31 cm^3^ and 32 cm^3^ in patients receiving tumor-specific therapy (p = 0.92). Of note, the overall mean CE volume was not significantly higher in the group of *MGMT*pos tumors when compared to *MGMT*neg tumors (in cm^3^, 28 versus 32, p = 0.48).

### Uni- and multivariate analyses

Multiple patient- and tumor-related factors were tested in a univariate fashion to assess a potential association with PFS or OS (Table 1). Out of the tested variables, a worse clinical status at first diagnosis as well as larger initial T2 volumes and CE volumes (all continuously evaluated) were associated with shorter PFS and OS. Frontal manifestation was associated with longer PFS than non-frontal manifestation, but not OS (Tables 1 and 3). *MGMT* promoter methylation was associated with longer OS, but not PFS (Table 1). In multivariate analyses for OS, clinical status and *MGMT* promoter methylation were associated with outcome (p < 0.01 and p = 0.01), but CE volume was not (p = 0.51, see Table 3). Univariate analyses were also performed exclusively for *MGMT*pos and *MGMT*neg tumors respectively. There were no statistically significant differences between the two cohorts (tested covariates: age, KPS, TMT, T2 tumor volume, CE volume, localization, multifocality). When comparing temozolomide-containing treatment regimens with RT alone, excluding patients from the BSC cohort, OS was significantly longer in the TMZ cohort: HR = 2.12, 95% CI 1.16–3.89, p = 0.01). This effect did not persist in multivariate analysis correcting for KPS, CE volume and *MGMT* status (KPS: p = 0.15; CE volume: p = 0.83; MGMT: p = 0.31; temozolomide vs. RT: p = 0.65),

**Table 3:** Multivariate analyses for progression-free and overall survival

Factor		PFS		
		HR	95% CI	*p*-value
**KPS** ^**x**^		0.97	0.95-1.0	< 0.01*
**CE volume** ^**x**^		1.01	1.0-1.02	0.22
**Frontal versus non-frontal localization**		2.05	1.11–4.15	0.03*
**Factor**		**OS**		
		HR	95% CI	*p*-value
**KPS** ^**x**^		0.96	0.94–0.99	< 0.01*
**CE volume** ^**x**^		1.0	0.99–1.01	0.51
***MGMT*** **promoter methylation status**		0.52	0.31–0.86	0.01*

KPS, Karnofsky Performance Status; CE volume, contrast-enhancing tumor volume; MGMT, O6-methylguanine-DNA methyltransferase; PFS, progression-free survival; OS, overall survival; ^x^ Continuously scaled. Significant p-values are highlighted with asterisks*

## Discussion

Treatment benefit in patients aged 80 years or older and diagnosed with glioblastoma is uncertain because possible toxic side effects of tumor specific therapy might outweigh any positive effects on survival in this vulnerable patient group. We investigated prognostic parameters and outcome in a retrospective cohort of patients with newly diagnosed glioblastoma aged 80 years or older and stratified the cohort according to the *MGMT* promoter methylation status as proposed by previous publications [10, 22]. In patients with *MGMT*pos glioblastoma, tumor-specific therapy comprising temozolomide was associated with a more than 2-fold increase in OS time when compared to BSC. In this subgroup, prognosis was especially favorable in patients with good clinical status and no polypharmacy. In patients with *MGMT*neg tumors, there was no differential OS benefit of tumour-specific therapy versus BSC (Fig. 2). These findings were confirmed in a matched-pair analysis (Fig. 3).

These results are important because they explicitly investigate the treatment response in the oldest-old patients with glioblastoma. While these results still need to be confirmed in prospective studies, they can already provide some guidance for clinicians caring for this patient group. An example for how oldest-old patients with glioblastoma were omitted from studies so far was the pivotal, randomized, phase 3 study on radiochemotherapy with concomitant and adjuvant temozolomide in patients with newly diagnosed glioblastoma [12]. This study defines the standard of care to this day, but excluded patients aged 70 years or older. The study found that the benefit of radiochemotherapy was pronounced in younger patients while the effect diminished after the age of 65 years [12]. In patients aged 60–70 years another prospective trial failed to demonstrate outcome differences between patients treated with temozolomide alone, hypofractionated or standard radiotherapy alone [10]. Patients aged 70 years or older and treated with standard radiotherapy (60.0 Gy in 2.0 Gy fractions over 6 weeks) surprisingly showed shorter overall survival (OS) than patients treated with hypofractionated radiotherapy (34 Gy in 3.4 Gy fractions over 2 weeks) or temozolomide treated with 200 mg/m^2^ in a 5/28 schedule [10]. These results highlight the vulnerability of the elderly when treated aggressively. Temozolomide monotherapy proved to be especially beneficial in patients with methylated *MGMT* promoter in this study.

There is some evidence in the literature comparing tumor-specific therapy and BSC in terms of OS in patients aged 70 years or older. A prospective, multicenter study established that radiotherapy was associated with better survival than palliative care only without compromising quality of life or neurocognitive function [18]. Prospective trials in even older patients are lacking. A retrospective study in patients with glioblastoma aged older than 80 years demonstrated extremely poor outcome overall with a median survival time of 4.1 months after diagnosis. Here, tumor-specific treatment was associated with prolonged survival compared to best supportive care (BSC). Unfortunately, the study did not include tumor volumes or the methylation status of the *MGMT* promoter region [12, 23, 34].

Whereas most patients who received therapy and had *MGMT*pos tumors were treated with temozolomide, almost all patients with *MGMT*neg tumors were treated with hypofractionated radiotherapy. Since there was no survival difference between radiotherapy and BSC in *MGMT*neg tumors, treatment benefit of hypofractionated radiotherapy appears questionable in *MGMT*neg patients aged 80 years or older.

It has been described that frailty is associated with worse outcome in elderly glioblastoma patients [35]. In this study we used KPS, TMT and polypharmacy as surrogate parameters for frailty. We found that KPS was high and polypharmacy was low in the subgroup of patients who lived > 18 months, suggesting that these patients were less frail.

Side effects of temozolomide are infrequent but can occur and lead to therapy discontinuation. The probability of severe adverse effects seems to increase with age [10, 22, 33, 36, 37]. In our cohort, the overall rate of grade 3 toxicities was 15% and within the range of previously published studies in elderly patients [10, 22]. Treatment-related toxicity seems to be acceptable even in the oldest-old and temozolomide is relatively easy to administer and monitor.

This study is limited by its retrospective design. A selection bias for decision against therapy must be assumed. This was in part accounted for by matched-pair analyses and should have played a systemic role in *MGMT*pos and *MGMT*neg patients, not only in one cohort. The number of patients receiving radiochemotherapy was small in our study. We therefore cannot make assumptions on the combined efficacy of radiochemotherapy as opposed to temozolomide alone in elderly *MGMT*pos patients. Another limitation is the lack of standardized surveys on neurocognition. Previous prospective studies have demonstrated that radiotherapy with a total dose of 50 Gy in elderly patients was not associated with significant differences in cognition or quality of life. In our study, most patients received hypofractionated radiotherapy or temozolomide. Accordingly, a significant treatment-induced decline in neurocognitive function in our cohort is unlikely to have occurred.

In summary, our data underlines the necessity of obtaining tissue and determining *MGMT* status in the elderly through biopsy if tumor resection is not safely feasible. Considering the low complication rates and survival benefit associated with the treatment, temozolomide seems to be a potent option for oldest-old patients with methylated *MGMT* promoter. We even observed survival times > 18 months in patients with methylated *MGMT* promoter and low frailty.

## Electronic supplementary material

Supplementary material 1

## Data Availability

Clinical and molecular data on all patients are anonymized and stored in local data bases secured by passwords.
