# Supplementary material for: Treatment benefit in patients aged 80 years or older with biopsy-proven and non-resected glioblastoma is dependent on MGMT promoter methylation status
Source: J Neurooncol. 2023 Jun 8;163(2):407–15. doi: 10.1007/s11060-023-04362-y (PMC10322768; doi:10.1007/s11060-023-04362-y)
Supplement: Supplementary file 1 — Supplementary material 1 [file 11060_2023_4362_MOESM1_ESM.docx]

# **Supplementary material**

Suppl. Table 1. Reasons for therapy discontinuation

| **Reason for therapy discontinuation** | **Temozolomide alone (n=22)** | **Radiotherapy**  **(n=23)** | **Radiochemotherapy**  **(n=7)** |
| --- | --- | --- | --- |
| Adverse events, n (%) | 4 (18) | 3 (13) | 1 (14) |
| Tumor progression, n (%) | 10 (46) | 3 (13) | 2 (29) |
